# Chronic Kidney Disease and the Aging Population

**Published:** 2014-02-01

**Authors:** M. Tonelli, M. Riella

**Affiliations:** 1 *University of Alberta. Alberta, Canada*; 2 *Catholic University of Parana, Curitiba, Brazil*

The proportion of older people in the general population is steadily increasing worldwide, with the most rapid growth in low- and middle-income countries [1]. This demographic change is to be celebrated, because it is the consequence of socioeconomic development and better life expectancy. However, population aging also has important implications for society—in diverse areas including health systems, labor markets, public policy, social programs, and family dynamics [2]. A successful response to the aging population will require capitalizing on the opportunities that this transition offers, as well as effectively addressing its challenges.

Chronic kidney disease (CKD) is an important public health problem that is characterized by poor health outcomes and very high health care costs. CKD is a major risk multiplier in patients with diabetes, hypertension, heart disease and stroke—all of which are key causes of death and disability in older people [3]. Since the prevalence of CKD is higher in older people, the health impact of population aging will depend in part on how the kidney community responds. 

March 13, 2014 will mark the celebration of the 9^th^ World Kidney Day (WKD), an annual event jointly sponsored by the International Society of Nephrology and the International Federation of Kidney Foundations. Since its inception in 2006, WKD has become the most successful effort to raise awareness among policymakers and the general public about the importance of kidney disease. The topic for WKD 2014 is “CKD in older people.” This article reviews the key links between kidney function, age, health and illness—and discusses the implications of the aging population for the care of people with CKD.

## EPIDEMIOLOGY OF AGING

**Figure 1:**
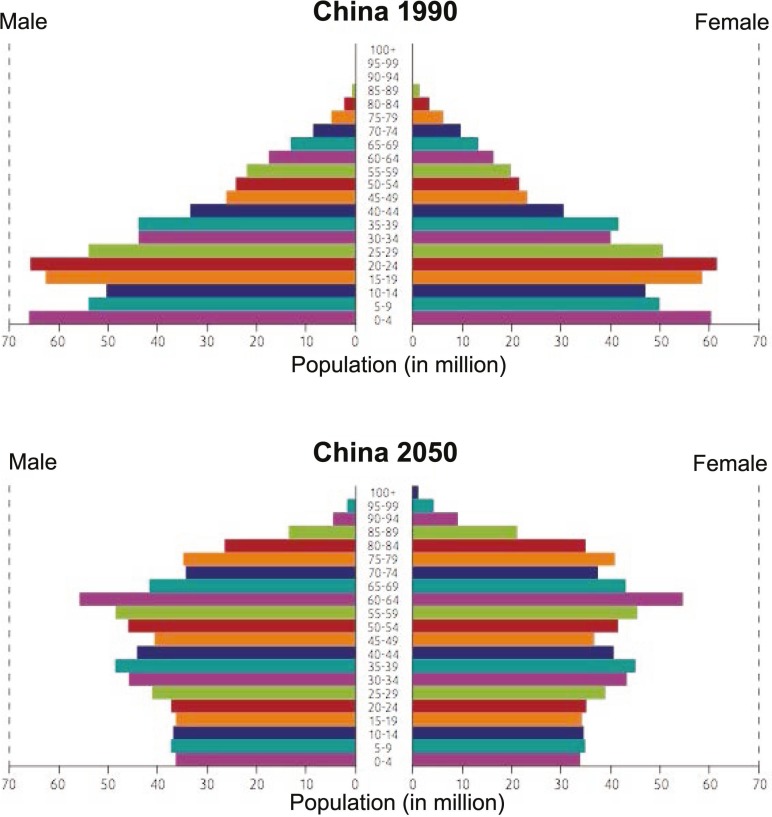
Changing age distribution in general population of China, 1990–2050. Reproduced from WHO document number WHO/DCO/WHD/2012.2.

The key drivers of population aging are socioeconomic development and increasing prosperity—which result in lower perinatal, infant and childhood mortality; lower risk of death in early adulthood due to accidents and unsafe living conditions; and improving survival of middle-aged and older people due to chronic disease. The resulting increases in life expectancy (together with the lower birth rates that typically accompany socioeconomic development) mean that older people account for a larger proportion of the general population [1]. The extent of the resulting changes in population characteristics can be startling, especially for developing countries (Fig 1).

In contrast to the situation even two generations ago, people can expect to live for many years after the usual retirement age. For example, the UK men and women aged 65 years in 2030 can expect to live until age 88 and 91 years, respectively [4]. Predicted life expectancy for today’s children is controversial, but experts estimate that 50% of the UK children born in 2007 will live to at least 103 years [4]. Although it is clear that people are living longer, it is uncertain how much of the increased life expectancy will translate into years of good health. These demographic changes have dramatic potential implications for conditions such as CKD, for which the prevalence increases with age.

## CKD IS COMMON IN OLDER PEOPLE AND ITS PREVALENCE INCREASES IN PARALLEL WITH AGE

It has been known for decades that estimated glomerular filtration rate (eGFR) declines in parallel with age [5]. The prevalence of CKD among females in the Chinese general population increases from 7.4% among those aged 18–39 years to 18.0% and 24.2% among those aged 60–69 and 70 years, respectively [6]. Relative increases in the prevalence of CKD with age are equally striking for populations in the US, Canada and Europe [7-9], although there are between-country differences in the absolute prevalence. 

At older ages, an increased proportion of prevalent CKD cases has low eGFR alone (as compared to albuminuria alone, or both low eGFR and albuminuria) [10]. Although this might suggest that many older people with CKD can expect lower rates of kidney function loss, available data are inconclusive—and current knowledge does not allow clinicians to reliably distinguish between those whose CKD will and will not progress. 

As for other age groups, the incidence of dialysis-dependent kidney failure has steadily increased among older people over the last few decades; in the US, a 57% age-adjusted increase in the number of incident octogenarians and nonagenarians was noted between 1996 and 2003 alone [11]. Despite this increase, patients aged >80 years are still less likely to initiate dialysis than those aged 75–79 years—although a large recent study suggested that the risk of developing very low eGFR (<15 mL/min/1.73 m^2^) is similar for older and younger adults [12]. It is uncertain whether this discrepancy is due to between-age differences in the true rate of progressive kidney function loss, the risk of death due to competing causes, patient views about dialysis, or physician practices [12, 13]. Regardless of the explanation, the aging population will likely lead to continued increases in the number of older people with severe CKD.

## CKD IS HARMFUL BUT TREATABLE IF PATIENTS AT RISK ARE IDENTIFIED

Like younger people, older people with advanced CKD are at increased risk of death, kidney failure, myocardial infarction and stroke compared to otherwise similar people with normal or mildly reduced eGFR [14, 15]. Although death is by far the most common of these adverse outcomes, this does not mean that older patients with clinically relevant CKD cannot benefit from timely specialist referral. 

With appropriate management, patients with advanced CKD (regardless of age) may benefit from slower loss of kidney function (potentially preventing kidney failure), better control of metabolic consequences such as acidosis, anemia and hyperphosphatemia, lower risk of cardiovascular events, and (for those who are interested in renal replacement) a more informed choice of renal replacement modality, including timely creation of vascular access [16]. The aging population will likely lead to continued increases in the number of older people who might require such referral, which should be considered in assessments of future nephrology workforce capacity.

## DIALYSIS CAN BENEFIT OLDER PEOPLE WITH KIDNEY FAILURE

In developed countries, the default management strategy for older people with kidney failure appears to have shifted from conservative management to initiation of dialysis [17] On average, life expectancy after initiation of dialysis is relatively short for older patients; median survival among incident US dialysis patients aged 80–84 years is 16 months—and is only 12 months among those aged 85–89 years [11]. At the same time, these median statistics reflect a bimodal distribution of survival time in older dialysis patients; although a large proportion die within six months of commencing dialysis, a substantial minority may live for years. This heterogeneity in mortality appears to be driven by differences in baseline comorbidity. For example, analyses of a small UK cohort of people with advanced kidney failure suggested that initiation of dialysis was not associated with increased survival for those aged >75 and with two or more comorbidities [18, 19]. Similarly, the presence of two to three comorbid conditions in US dialysis patients aged >65 years was associated with substantially increased mortality compared to those in better health [11]. When functional status is lower at baseline, initiation of dialysis often signals the onset of further declines; among 3702 nursing home residents initiating dialysis, 58% had died and 87% had experienced additional loss of function at one year [20]. Although available data have limitations, quality of life appears reasonable among selected older dialysis patients—and can remain stable despite moderate or high levels of comorbidity [21, 22].

These data suggest that dialysis is an appropriate treatment option for well-informed older patients with kidney failure—especially, for those with good baseline quality of life. On the other hand, the very poor outcomes experienced in those with more comorbidity or lower functional status at baseline clearly demonstrate that dialysis does not improve clinical outcomes for all older people with kidney failure—and that good clinical judgment and careful communication will be increasingly required as the general population continues to age.

## KIDNEY TRANSPLANTATION CAN ALSO BENEFIT OLDER PEOPLE WITH KIDNEY FAILURE

It is generally accepted that older age alone does not preclude kidney transplantation in otherwise suitable candidates. However, older patients with kidney failure are more likely to have absolute and relative contraindications to transplantation, and are less likely to be placed on the kidney transplantation waiting list. Unsurprisingly, patient and graft 5-year survival probabilities are lower among US kidney transplant recipients aged ≥65 years as compared to those aged 35–49 years (patient: 67.2% *vs* 89.6%; graft: 60.9% *vs* 75.4%, respectively) [23]. In addition, older people who are potential kidney transplant recipients face several potential disadvantages compared to their younger counterparts (Box 1).

**Box 1:** Unmet needs for kidney transplantation in older CKD patients

Organ shortage
Paucity of live donors
Organ allocation policies that appropriately weight likelihood of benefit from transplantation as well as chronological age
Ensuring appropriate referral of potentially suitable older recipients for transplantation assessment
Ethical concerns about offering a kidney to an older patient versus a younger one
Optimal immunosuppressive regimen

Adapted from reference 29

Nonetheless, transplantation appears to reduce mortality among patients of all ages. For example, among those aged 74 years, receiving a deceased donor transplant was associated with a hazard ratio of mortality of 0.67 (95% CI: 0.53–0.86) as compared to remaining on dialysis [23]. Use of expanded criteria deceased donors [24, 25], as well as more liberal use of older living donors [26], also appear to reduce mortality among older people with kidney failure, as compared to similar patients who remain on the transplant waiting list (Box 2). These latter two strategies are especially appealing for use in developing countries, where growth in the prevalence of older people has been most pronounced. However, because transplant surgery itself temporarily increases the risk of death, the mortality benefits associated with kidney transplantation (regardless of donor type) are restricted to those with reasonable baseline life expectancy and without dramatically increased perioperative risk [27].

**Box 2:** Meeting the growing demand for kidney transplantation in older CKD patients

Preferential transplantation of organs from older donors to older recipients
Enlarge the donor pool by accepting expanded criteria donors: ≥60 years old or ≥50 with any of the following two conditions: history of hypertension, serum creatinine ≥1.5 mg/dL or death due to cerebrovascular accident.
“Old for old”: preferentially using kidneys from older living donors for older recipients
Transplanting two marginal kidneys instead of one

Adapted from reference 29

## RESEARCH NEEDS

Although much is known about chronic kidney disease in older populations, a great deal remains to be learned. Many trials of therapies for CKD have excluded older patients [28]—and most do not provide guidance on how to manage comorbidities that often accompany CKD but may lead to competing therapeutic priorities. More information is needed on how to accurately identify people who will progress to kidney failure—and among these, the subset that can expect reasonable life expectancy and quality of life if they opt for dialysis treatment. Future studies should test new ways to communicate information about the risks and benefits of dialysis (as compared to conservative management), to facilitate informed patient decisions. Above all, we need more studies that demonstrate how to optimize quality of life and manage symptoms in elderly people with CKD—including those who have chosen conservative management. 

## THE WAY FORWARD

The aging of the general population means that older people now account for a much greater proportion of patients with or at risk for kidney disease and kidney failure. The tremendous clinical heterogeneity within this population indicates the need for more discerning management. Chronological age alone will not be sufficient as the basis for clinical decisions, and a more nuanced approach is required—based on the comorbidities, functional status, quality of life and preferences of each individual patient. Clinicians can be reassured that dialysis and kidney transplantation can increase life expectancy—and will allow reasonable quality of life in selected older people with kidney failure. Perhaps more importantly, clinicians, patients and their families can be comforted by the knowledge that timely specialist evaluation can help to improve outcomes and reduce symptoms in older people with advanced kidney disease—whether they have selected conservative management or dialysis as their treatment plan.

## Footenote

This Editorial may also be published by other medical or biomedical journals.
